# Challenges to the development of vaccines to hepatitis C virus that elicit neutralizing antibodies

**DOI:** 10.3389/fmicb.2014.00329

**Published:** 2014-07-03

**Authors:** Heidi E. Drummer

**Affiliations:** ^1^Viral Fusion Laboratory, Centre for Biomedical Research, Burnet InstituteMelbourne, VIC, Australia.; ^2^Department of Microbiology, Monash UniversityClayton, VIC, Australia; ^3^Department of Microbiology and Immunology, The Peter Doherty Institute for Infection and Immunity, The University of MelbourneParkville, VIC, Australia.

**Keywords:** glycoprotein E2, neutralizing antibody, CD81, immune evasion, viral entry

## Abstract

Despite 20 years of research, a vaccine to prevent hepatitis C virus (HCV) infection has not been developed. A vaccine to prevent HCV will need to induce broadly reactive immunity able to prevent infection by the 7 genetically and antigenically distinct genotypes circulating world-wide. HCV encodes two surface exposed glycoproteins, E1 and E2 that function as a heterodimer to mediate viral entry. Neutralizing antibodies (NAbs) to both E1 and E2 have been described with the major NAb target being E2. The function of E2 is to attach virions to host cells via cell surface receptors that include, but is not limited to, the tetraspanin CD81 and scavenger receptor class B type 1. However, E2 has developed a number of immune evasion strategies to limit the effectiveness of the NAb response and possibly limit the ability of the immune system to generate potent NAbs in natural infection. Hypervariable regions that shield the underlying core domain, subdominant neutralization epitopes and glycan shielding combine to make E2 a difficult target for the immune system. This review summarizes recent information on the role of NAbs to prevent HCV infection, the targets of the NAb response and structural information on glycoprotein E2 in complex with neutralizing antibodies. This new information should provide a framework for the rational design of new vaccine candidates that elicit highly potent broadly reactive NAbs to prevent HCV infection.

## ARTICLE

The most cost effective means of controlling infectious disease is through vaccination, however, a prophylactic or therapeutic vaccine for HCV is not available. HCV is a small, enveloped positive sense RNA virus within the Flaviviridae family. HCV is classified into seven major genotypes that display up to 33 % nucleotide variation and >100 subtypes that display up to 20% nucleotide variation. The genotypes of HCV have a geographical distribution with genotypes 1–3 prevalent world-wide. Individuals infected with HCV harbor a swarm of closely related viruses referred to as a quasispecies. The degree of sequence variation observed for HCV exceeds that for HIV and influenza, posing a major challenge to vaccine development. Essential to the success of an HCV vaccine will be an ability to confer broad protection against the circulating strains of HCV.

## THE ROLE OF ANTIBODY IN HCV INFECTION

The effectiveness of all current licensed viral vaccines relies on the production of neutralizing antibody (NAb; Lambert et al., 2005). Strong evidence now exists that NAbs play a major role in clearance of HCV infections. Longitudinal analysis of HCV infection cohorts reveals that broadly specific NAb (brNAb) elicited early in infection correlates with viral clearance (Lavillette et al., 2005; Pestka et al., 2007; Dowd et al., 2009). By contrast, people who failed to make NAbs progressed to chronic infection. Sequence analysis of the structural region during chronic infection reveals that the development of a NAb response correlates with sequence evolution, likely to be a result of immune escape (von Hahn et al., 2007; Liu et al., 2010). In a single case study, Raghuraman et al. (2012) examined the NAb and cellular immune responses in a patient with long term chronic HCV who spontaneously cleared their virus after 62 weeks of infection. Viral clearance correlated with the appearance of NAb at 48 weeks and a reversal of T cell exhaustion. Neutralizing antibodies also play an important role in people who have previously cleared their HCV infection but become infected on reexposure to HCV. Neutralizing antibodies were detected during the acute phase of infection in 60% of reinfected participants who went on to clear their reinfection but was not detected in 2 patients who progressed to chronic infection (Osburn et al., 2010). Overall, the findings of these studies suggest that the development of a NAb response during the early phase of acute infection is a strong correlate of viral clearance. Consistent with these observations, passive transfer of broadly neutralizing monoclonal or polyclonal NAbs to experimental animals protects them against viral challenge (Law et al., 2008; Vanwolleghem et al., 2008; Morin et al., 2012). However, administration of a sterilizing dose of antibody must precede infection, otherwise the antibody is not effective at preventing infection, but can reduce viral loads (Meuleman et al., 2008; Morin et al., 2012).

## HCV GLYCOPROTEINS E1 AND E2

Hepatitis C virus encodes two glycoproteins, E1, and E2 that are cleaved from the viral polyprotein by signal peptidases. E1/E2 function as a heterodimer to mediate viral entry. E2 mediates attachment to cellular receptors CD81 and scavenger receptor class B type 1 (SR-B1) via sequences within its RBD (Pileri et al., 1998; Scarselli et al., 2002). The functional properties of E1/E2 and the ability of E1/E2-specific antibodies to prevent viral entry can be studied using retroviruses pseudotyped with E1/E2 (HCVpp; Bartosch et al., 2003; Drummer et al., 2003; Hsu et al., 2003) or cell culture derived HCV (HCVcc) made by transfecting full-length HCV RNA into Huh 7 cells (Lindenbach et al., 2005; Wakita et al., 2005; Yi et al., 2006).

**FIGURE 1:**
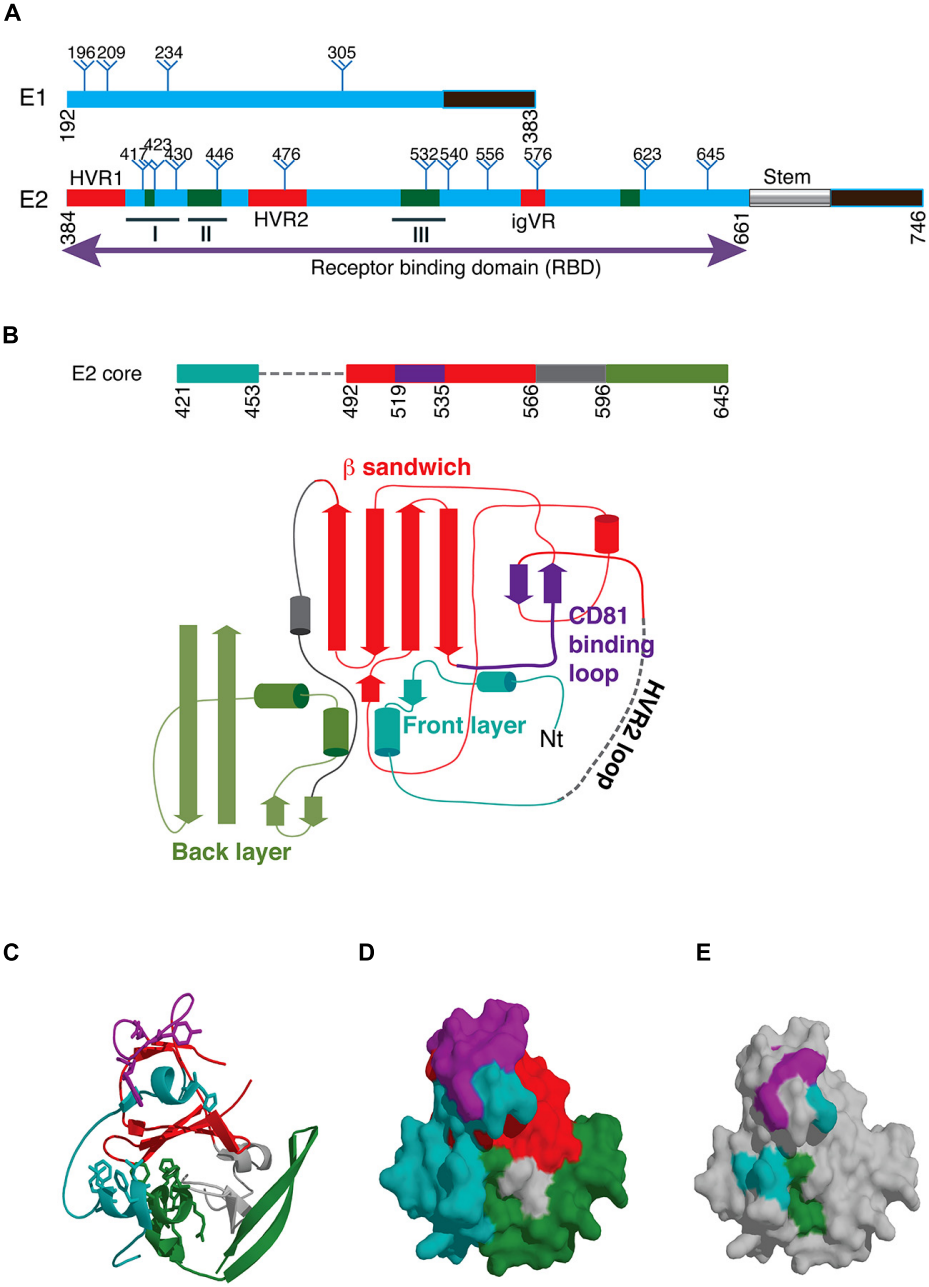
**(A)** Schematic of hepatitis C virus glycoproteins E1 and E2 showing the location of conserved glycosylation sites (trees). The transmembrane domains are shown in black and the E2 stem region is indicated with a cylinder. The location of hypervariable regions in E2 is shown in red and the three immunogenic epitopes designated epitopes I, II, and III are underlined. Regions involved in CD81 binding are shown in green with specific contact residues shown in **Table 1**. **(B)** Schematic of the E2 core domain according to the designations of Kong et al. (2013). **(C)** Structure of the E2 core domain colored according to **B**. **(D)** Surface representation of **C**. Purple and teal regions represent the neutralizing face of E2 and overlap with CD81 contact residues shown in **E**. **(E)** Surface representation of the E2 core domain with those residues involved in CD81 with their locations colored according to **B**.

*Glycoprotein E1* is essential for viral entry as HCVpp lacking E1 is non-infectious but its function in entry is unknown (Drummer et al., 2003). E1 contains a C-terminal transmembrane domain (TMD) that anchors the ectodomain to the virion and contains four or five glycosylation sites (**Figure 1A**). Relatively few NAbs have been described for E1 (Keck et al., 2004b; Meunier et al., 2008) suggesting it is a subdominant immunogen in natural infection. Glycoprotein E1 is essential for the correct assembly and stability of the E1/E2 heterodimer in HCVcc and allosterically modulates the structure of E2 and its ability to bind cellular receptors (McCaffrey et al., 2011; Wahid et al., 2013).

*Glycoprotein E2* comprises ∼ 11 largely conserved N-linked glycosylation sites and 18 conserved cysteines (**Figure 1A**). The receptor binding domain, residues 384–661 (RBD), folds independently of other E1/E2 sequences. The RBD is linked through a conserved C-terminal stem to the TMD (Drummer and Poumbourios, 2004; **Figure 1A**). The binding site for CD81, the major cellular receptor for all HCV strains, comprises highly conserved segments within the E2 RBD (Drummer et al., 2002; Roccasecca et al., 2003; Zhang et al., 2004; Drummer et al., 2006; Owsianka et al., 2006; **Figure 1A**). These regions of E2 interact with the large extracellular loop (LEL) of CD81 through Ile182, Asn184, Phe186, and Leu162 on the head subdomain (Higginbottom et al., 2000; Drummer et al., 2002).

## VARIABLE REGIONS OF THE E2 GLYCOPROTEIN

Located within the RBD are four variable regions (**Figure 1A**). The N-terminal hypervariable region 1 (HVR1) is 27 amino acids in length and resides outside the core domain of E2, and no structural information is available (Weiner et al., 1991; Kato et al., 1992). Despite the high degree of sequence variation, the overall basic charge of this region is preserved, possibly to maintain interactions with SR-B1 (Penin et al., 2001; Dao Thi et al., 2011). Deletion of HVR1 from the E2 RBD abolishes the interaction with SR-B1, while deletion of HVR1 in the context of HCVpp abolishes high-density lipoprotein (HDL)-mediated enhancement of viral entry, thereby increasing the effectiveness of NAb (Dao Thi et al., 2011). Originally, hypervariable region 2, HVR2, was described as a nine amino acid sequence (Kato et al., 1992) downstream of HVR1. Further analysis across different HCV genotypes suggested a more extensive area of variation (res. 461–481) flanked by conserved cysteine residues that form a surface exposed disulfide bonded loop, not essential for folding of the E2 RBD core (McCaffrey et al., 2007; Kong et al., 2013). Sequence identity within HVR2 ranges from 39% in genotypes 1a and b to 93% in genotype 5a (McCaffrey et al., 2007). An additional cysteine-flanked variable region (**igVR**, res. 570–580) was described that is relatively conserved within a genotype but exhibits a high degree of intergenotypic variation in both length (10–15 res) and sequence (McCaffrey et al., 2007). Deletion of either HVR2 or the igVR in the context of E1/E2 incorporated into HCVpp is not tolerated as E2 fails to form heterodimers with E1 and the resultant HCVpp are not infectious (McCaffrey et al., 2011). These data suggest that HVR2 and the igVR are essential to the virion incorporated E1/E2 structure. There is no evidence in the literature to suggest that HVR2 or the igVR are targets of the antibody response and so are unlikely to be under immune pressure. McCaffrey et al. (2007) have shown that HVR1, HVR2, and the igVR can be simultaneously deleted from the RBD without disrupting its native fold and receptor binding ability, indicating that they are outside a conserved core domain (McCaffrey et al., 2007). Analysis of sequence variation within patient isolates revealed a third hypervariable region (HVR3) within the sequence 434–450, that appears to be under immune selection pressure (Troesch et al., 2006; Torres-Puente et al., 2008).

## THE THREE DIMENSIONAL STRUCTURE OF THE E2 CORE DOMAIN

Recently, X-ray crystallography and electron microscopy have provided the first insights into the structure of glycoprotein E2. The structure provided by Kong et al. (2013) was obtained for an E2 core domain containing residues 412–645 that lacked two glycosylation sites at Asn448 and Asn576, and HVR2 residues 460–485 were replaced with a Gly-Ser-Ser-Gly linker (**Figure 1B**). The second structure was for an E2 core comprising residues 456–656 (Khan et al., 2014). The structures were largely similar and revealed that unlike its related glycoprotein E flavivirus counterparts HCV E2 does not have a three domain architecture reminiscent of other class II fusion proteins. Instead, E2 core adopts a compact globular immunoglobulin-like fold comprising a central β-sandwich surrounded by short front and back layers comprising loops, short helices and β sheets (Kong et al., 2013; Khan et al., 2014; **Figures 1B,C**). Verification that E2 adopts a compact globular fold when expressed as the complete E2 ectodomain was provided by electron microscopy (Kong et al., 2013). The immunoglobulin fold resembles domain III of the class II fusion proteins, the only common structural element with the alpha and flavivirus fusion proteins. In the core domain of HCV E2, 8 disulfide bonds were formed but many regions lacked regular secondary structure including the regions between residues 412–420 and 454–491 surrounding the truncated HVR2 region and a loop at 586–596 (Kong et al., 2013). Six of the N-linked glycans also were also largely disordered at Asn417, Asn423, Asn532, Asn540, Asn623, and Asn556 (Kong et al., 2013). The immunoglobulin sandwich is formed by 4 β strands that form an inner sheet and two solvent exposed β strands that comprise the outer sheet. A loop connects the inner sheet to the outer sheet and contains many of the key CD81 binding residues and is adjacent to the front layer where additional CD81 contact residues are found that are surface exposed (**Figures 1D,E**). The igVR is within a flexible region spanning 567–596 and the back layer is formed by two short α-helices and 4 β sheets.

## TARGETS OF THE ANTIBODY RESPONSE TO HCV

### ANTIBODY RESPONSE TO E1

Neutralizing monoclonal antibodies specific to E1 or epitopes that are targets of NAbs have been described although they are relatively limited in number. This may in part be due to the inability to express a recombinant form of E1 that is a known mimic for the structure of E1 in virions and the likelihood that E1 is largely occluded by E2 in virion incorporated heterodimers. Antibody H-111 binds an epitope at the N-terminus of E1, immunoprecipitates E1/E2 heterodimers and has cross-neutralizing potential (Keck et al., 2004b). Two MAbs isolated from a human who had cleared their genotype 1b infection, IGH505 and IGH526 recognize overlapping but distinct epitopes within the sequence 307–340, and neutralize genotypes 1a, 1b, 4a, 5a, and 6a viruses, but not 2b or 3a. Interestingly, only 2/31 MAbs isolated by Meunier et al. (2008) were specific to E1 neutralized virus suggesting that such antibodies may be rarely elicited. Additional epitopes recognized by human serum have been discovered using overlapping peptides or using mass spectrometry and include epitopes at 264–318 (Kachko et al., 2011) and 308–325 (Grollo et al., 2006).

### THE ANTIBODY RESPONSE TO E2

Thus far, two specificities of NAb elicited in natural infection and following vaccination with E2 derived immunogens have been identified. Neutralizing antibodies directed toward the N-terminal HVR1 are immunodominant in natural infection (Kato et al., 1993, 1994; Zibert et al., 1995). Immune serum raised to HVR1 has the ability to protect chimpanzees from experimental infection with a homologous strain of virus but are type-specific with limited ability to neutralize immune escape variants within the HVR1 sequence (Farci et al., 1996). As a result antibodies are believed to have a major role in driving immune escape (Edwards et al., 2012) and HVR1 has been proposed to be an immune decoy (Mondelli et al., 2001). Analysis of the evolution of HCV sequences in people infected with HCV reveals that in the absence of NAb activity, the HVR1 sequence remains stable but rapidly evolves under NAb pressure (Liu et al., 2010). Analysis of HVR1 specific antibody responses in HCV-infected people revealed that those who cleared virus spontaneously were more likely to have detectable anti-HVR1 antibodies in their serum within the first 6 months of infection (Zibert et al., 1997) and suggests that induction of HVR1 specific antibodies in some circumstances may favor viral clearance.

While HVR1 specific antibodies are largely type-specific, evidence that some anti-HVR1 antibodies can be cross reactive was obtained by vaccinating rabbits with a panel of HVR1 peptides (Shang et al., 1999). The subsequent antibody response was able to recognize diverse HVR1 sequences, capture HCV from patient serum and prevent HCV binding to cells. A monoclonal antibody specific to HVR1 was also shown to be cross reactive and contained conserved amino acids within its epitope (Li et al., 2001). While neutralization assays were not available at the time these studies were preformed the data suggest that HVR1 cannot be entirely discounted as a desirable component of an HCV vaccine.

The second major specificity of NAb is directed toward sequences involved in CD81 binding. Antibodies specific to the CD81 binding site located on the surface of E2 are frequently cross neutralizing due to the high degree of sequence conservation (Keck et al., 2004a; Johansson et al., 2007; Law et al., 2008). Residues involved in binding CD81 have been mapped by performing mutagenesis of E2 RBD E1/E2 and E1/E2 incorporated into HCVpp and is summarized in **Table 1**. A striking feature of these contact residues is that they are mostly aromatic and hydrophobic amino acids and they are highly conserved between genotypes or the substitutions are conservative; in the case of Trp437, Phe is more frequently observed in genotypes 1b and 2-7, and for residues Leu438 and Phe442 alternative amino acids are I, V or M and L, M or I, respectively. The major NAb specificities elicited toward E2 that block CD81 binding in infected humans recognize epitopes that contain amino acids within the regions spanning residues 411–428 and 429–448 and 523–549 and thus directly overlap with the regions of E2 involved in CD81 binding. These regions involved in CD81 binding overlap with neutralization epitopes and this area represents the neutralizing face of E2 (**Figures 1D,E**).

**Table 1:** Residues of E2 involved in binding to CD81.

Residue^a^	Epitope^b^	Alternative amino acids^c^	Reference
Trp420	I	–	Owsianka et al. (2006)
His421	I	–	Boo et al. (2012)
Trp437	II	Phe	Drummer et al. (2006)
Leu438	II	Ile,Val, Met	Drummer et al. (2006)
Leu441	II	–	Drummer et al. (2006)
Phe442	II	Leu, Met, Ile	Drummer et al. (2006)
Tyr527	III	–	Owsianka et al. (2006)
Trp529	III	Phe	Owsianka et al. (2006)
Gly530	III	–	Owsianka et al. (2006)
Asp535	III	–	Owsianka et al. (2006)
Y^613^RLWHY^d^		–	Roccasecca et al. (2003)

*^a^Analysis was performed on genotype 1a isolates. ^b^Location of amino acid within an antigenic region. ^c^Alternative amino acids observed in other isolates of HCV. A dash indicates conserved.^d^Data derived by replacing this region with SAASAS.*

A subset of antibodies directed to these regions can bind to their epitopes in the context of synthetic peptide analogs of 411–428 (epitope I) and 429–448 (epitope II; **Figure 1A**). Examples of human monoclonal NAbs that recognize these regions include HCV1, and 95-2 (epitope I), and 84-1, 84-25, 85-26 and 84-27 (epitope II). Antibodies that bind their epitope within the 523–549} sequence, referred to as epitope III, have also been described and include e20, (1:7) and A8 but appear to be conformation dependent (Allander et al., 2000; Johansson et al., 2007; Mancini et al., 2009; Edwards et al., 2012). Another subset of antibodies recognize discontinuous epitopes containing amino acids from one or more of these regions and only bind folded E2 in the context of the recombinant RBD or virion incorporated E2 such as CBH-5, CBH-7, AR3A, AR3B, AR3C, AR3D, and Fab e137 (Hadlock et al., 2000; Law et al., 2008; Owsianka et al., 2008; Perotti et al., 2008; Edwards et al., 2012).

Recently, X-ray crystal structures have been determined for neutralizing MAbs in complex with synthetic peptides analogs of epitopes I and II and of the region encompassing epitope III in the context of the E2 core domain. The structures reveal important information about the conformation of these epitopes, the mode of binding and the presence of a neutralizing face on the surface of the E2 molecule.

### EPITOPE I

The region encompassing epitope I is absent from the available structures of the E2 core domain but two structures of MAbs in complex with synthetic peptides reveal important structural information about this region. The murine antibody AP33 was first discovered in 2001 and is a brNAb specific to epitope I (Owsianka et al., 2001, 2005). Its binding site comprises a discontinuous sequence within this region and includes amino acids Leu413, Asn415, Gly418, and Trp420 with contribution by Asn417. These amino acids are highly conserved between HCV isolates and explain its broad cross reactivity (Tarr et al., 2006). However, this specificity of antibody does not appear to be frequently elicited in patients with HCV as it was detected in only 2.5% of serum samples collected from acute and chronic phases of HCV (Tarr et al., 2007). The X-ray structure of AP33 with epitope I reveals that the peptide forms a type 1 β-hairpin structure that is sandwiched between the heavy and light chain of antibody. Hydrogen bonds formed with Asn416 and Gly418 and hydrophobic interactions between the side chains of Leu413 and Trp420 form the major interactions and residues from all CDR loops except L2 are involved in binding to the peptide (Kong et al., 2012a).

**FIGURE 2:**
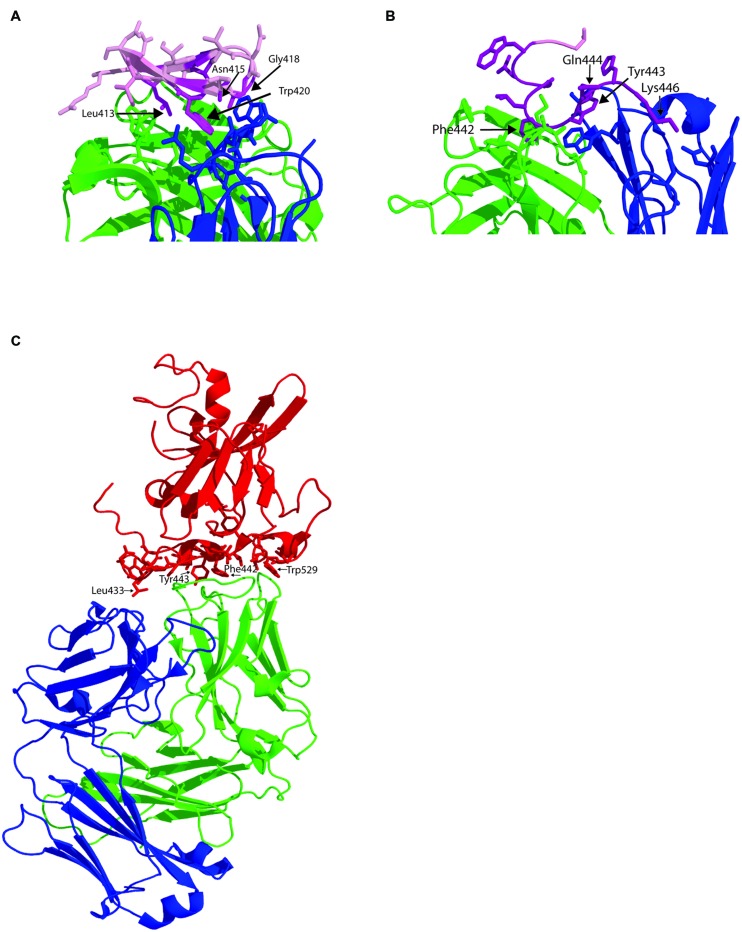
**Structure of human MAbs in complex with their epitopes. (A)** Structure of the paratope of MAb HCV1 in complex with synthetic peptide spanning residues 412–423 within E2 epitope I (pink). Amino acids side chains within the paratope in contact with the antibody epitope are shown as sticks. The antibody heavy chain is colored green and the light chain is in blue and major contact residues within the peptide shown as purple sticks. **(B)** Structure of the paratope of HC84-1 with its epitope spanning 435–446. Labeled as for **A**. **(C)** Structure of the E2 core domain in complex with neutralizing antibody AR3C. E2 is colored in red with the side chains of the amino acid in contact with the antibody shown as sticks. Heavy chain is green and light chain is blue.

The human MAb HCV1 is specific to epitope I and is able to neutralize genotype 1a, 1b, 2b, 3a, and 4a and binds its epitope at nanomolar affinity (Broering et al., 2009). Administration of HCV1 to chimpanzees protects against HCV infection and can be used to treat acutely infected chimpanzees (Morin et al., 2012). As a result of these promising results, HCV1 is currently being evaluated in clinical trials for its ability to prevent reinfection of the liver following transplantation. The major contact residues are Leu413 and Trp420 (Broering et al., 2009) although NAb escape mutants can be selected that possess the mutation Asn415Asp/Lys. The crystal structure of HCV1 in complex with a peptide containing HCV residues 412-423 again reveals a type 1 β hairpin structure for the peptide in contact with the heavy chain CDR2 and CDR3 loops and the CDR3 loop of the light chain (Kong et al., 2012b; **Figure 2A**). The ability of HCV1 to mediate broad neutralization is further explained by the conservation of its contact residues and the solvent accessibility of this region on the surface of E2. Additional alanine scanning performed in the context of E1/E2 heterodimer revealed that Asn415 and Gly418 were essential for HCV1 binding because these residues are critical to the formation of the β hairpin turn and the conformation of epitope I. Mutagenesis studies revealed that substitution of Asn415 with glutamine, glutamate or lysine preserved the β turn architecture and maintained viral fitness while significantly reducing the effectiveness of HCV1 to neutralize virus, and thus provides a potential mechanism for *in vivo* escape (Kong et al., 2012b).

### EPITOPE II

The region encompassing epitope II overlaps extensively with key CD81 contact residues (Drummer et al., 2006) and brNAb that bind this region have only recently been described (Law et al., 2008; Keck et al., 2012). Antibodies to this region are particularly interesting as passage of cell culture derived virus with a neutralizing amount of antibody fails to select for escape mutants suggesting that fit viruses with escape mutations cannot be selected (Keck et al., 2012). Two antibodies, 84-1 and 84-27 recognize a synthetic peptide analog and their crystal structures have been determined (Krey et al., 2013). In both cases, the peptide adopts the same conformation suggesting it reflects that observed in the native structure of E2, and not an induced fit conferred by the antibody. The peptide adopts a 1.5 α-helical turn spanning Trp437-Phe442 with Tyr438-Lys446 adopting an extended conformation. In the case of 84-1, E2 amino acids Leu441 and Phe442 make extensive hydrophobic contact with the antibody heavy chain that is further stabilized by a hydrogen bonding network that includes Lys446 and Trp443 (**Figure 2B**). Analysis of the Los Alamos HCV database revealed that Thr435, Gly436, Ala439, Leu441, and Tyr443 are highly conserved while Gln444 and His445 are less conserved. The key contact residue Phe442, conserved in 60% of sequences, can only be replaced with bulky hydrophobic amino acids. The conservation of Leu441 and Phe442 suggest a strict structural constraint at this position to maintain glycoprotein function, possibly explaining why neutralization escape mutants could not be selected with these antibodies. This immunogenic domain of E2 overlaps with HVR3 and suggests that the region is under immune selection pressure suggesting that some antibodies to this region, elicited in natural infection, may not be able to prevent immune escape *in vivo*, as was observed for 84-1 *in vitro*.

### EPITOPE III

The E2 structure provided by Kong et al. (2013) was obtained by co-crystallization of E2 with neutralizing human MAb AR3C whose epitope contains residues from epitope III at positions 530, 535, 538, and 540 and one residue from epitope I at position 424 (Law et al., 2008; **Figure 2C**). In the co-crystal structure, AR3C buries 828 Å of E2 surface area and 161 Å of E2 glycan surface area and additional points of contact with E2 were revealed (Kong et al., 2013). 86% of the buried E2 surface comprises residues that are 80–100% conserved across all E2 sequences. The epitope of AR3C comprises most of the front layer, some amino acids between 421–446 and the CD81 binding loop. The majority of contacts with E2 are mediated by the heavy chain CDR3 (44% of total buried surface). The binding site for AR3C overlaps directly with the CD81 binding site and is a surface exposed, hydrophobic region, relatively unobscured by carbohydrate (Kong et al., 2013). A vaccine with the ability to elicit such antibody specificities is therefore highly desirable as they are likely to have broad neutralization potential.

## WHY HAVE VACCINES FOR HCV NOT BEEN DEVELOPED?

Unlike its flavivirus counterparts, expression of the HCV glycoproteins E1 and E2 does not result in the secretion of subviral particles, the component of all flaviviral vaccines. The ability to culture HCV was developed in 2005 but remains limited to a handful of isolates and titres remain low making large-scale production of an inactivated vaccine unlikely. Nevertheless, Akazawa et al. (2013) have successfully demonstrated that sufficient HCVcc can be produced from cell culture and the inactivated HCVcc were used to immunize mice. The antibodies were able to neutralize genotypes 1a, 1b, and 2a viruses and prevented the infection of human liver transplanted onto uPa-SCID mice but only at the lowest homologous challenge dose of virus (Akazawa et al., 2013). *In vivo* heterologous neutralization was not examined. The immune serum from mice vaccinated with the inactivated HCVcc particles were more efficient at neutralizing homologous virus than immune serum from mice vaccinated with recombinant E2 alone or recombinant E1 and E2, suggesting intact particles are likely to be more immunogenic than the isolated recombinant glycoproteins.

Only one HCV vaccine candidate aimed at eliciting NAb has been tested in humans. The vaccine initially developed by Chiron Corporation was based on recombinant HCV E1 and E2 glycoproteins purified from mammalian cells as a non-disulfide linked heterodimer. In preclinical studies conducted in chimpanzees, the vaccine induced high titres of E1 and E2 specific antibodies and prevented five chimpanzees from becoming infected with a homologous challenge of virus and protection correlated with the presence of high titre anti-E2 antibodies (Choo et al., 1994). Challenge of the chimpanzees with a closely related heterologous virus strain resulted in infection in all cases; all but one vaccinee did not progress to chronic infection, suggesting the vaccine does induce a degree of protective but not sterilizing immunity (Houghton and Abrignani, 2005).

The recombinant E1/E2 vaccine was trialed in healthy human volunteers and adjuvanted with MF59. The initial results of this trial revealed that 15/41 subjects had antibody reactive to E1 region 313-327, 21/41 subjects had anti-HVR1 specific antibodies, 23/41 had epitope I reactive antibodies, and 13/41 had epitope II reactive antibodies, where reactivity was defined as a greater than twofold higher optical density compared to pre-immune or placebo samples (Ray et al., 2010). Nine, 11, 10, and 5 samples with E1, HVR1, epitope I, or epitope II reactive antibodies, respectively, neutralized virus, although it is not entirely clear what level of neutralization was achieved. The higher proportions of epitope I reactive antibodies compared to that reported in natural infection is a promising outcome of this vaccine trial if it can be correlated with an increase in cross-neutralizing activity in these sera. In a subsequent study, using a more extensive panel of HCVcc from each of the 7 genotypes revealed that of the three subjects tested, one subject elicited sera with the ability to mediate ∼25 to 80% neutralization of HCVcc at a 1/50 dilution of serum with the highest neutralization observed against genotype 6 and the lowest to genotype 7 (Law et al., 2013). These results should provide encouragement to the field of vaccine research that cross neutralizing antibodies can be elicited by recombinant E1/E2 based vaccines and warrant further study. A major limitation of the studies described above are the relatively low titres of NAbs elicited, their limited cross neutralizing potential, and their modest level of neutralization. A number of mechanisms whereby the HCV glycoproteins can evade the immune response have been illuminated and suggest that alternative approaches to vaccine design may be required, similar to the approach currently being used for HIV and influenza (Nabel, 2012).

## INTERFERENCE OF NEUTRALIZATION BY VARIABLE REGIONS OF E2

The three variable regions of E2 have been implicated in immune evasion by HCV. HVR1 contains the epitopes of NAbs that block E2 binding to SR-B1 but not to CD81 (Sabo et al., 2011). Instead, HVR1 antibodies can allosterically shield the CD81 binding site and conserved NAb epitopes therein providing an additional mechanism of neutralization escape (Bankwitz et al., 2010). Roccasecca et al. (2003) examined the effect of chimerization of HVR1 and HVR2 in closely related strains of HCV and the effect of HVR1 deletion on CD81 binding. The efficiency of CD81 binding was found to be strain dependent and deletion of HVR1 enhanced CD81 binding when performed in the context of recombinant E2 RBD and recombinant CD81 LEL and RBD binding to CD81 on the surface of Molt-4 cells suggesting that HVR1 may at least partially occlude the CD81 binding site. When HVR1 or HVR2 was swapped between E2 strains, no difference in CD81 binding was observed. However, when both HVR1 and HVR2 of strain H were interchanged with those of the N2 strain, a fourfold reduction in CD81 binding was observed. The reciprocal chimera resulted in an increase in CD81 binding (Roccasecca et al., 2003). These results suggest that CD81 binding is modulated by the presence of HVR1 and the sequence of HVR2 implying that these regions are flexible surface exposed domains that may provide a means to shield the underlying CD81 binding site, occluding it from immune surveillance.

The effect of the variable regions on CD81 binding was further explored by McCaffrey et al. (2007). In this study, HVR1, HVR2, and the igVR were deleted from the E2 RBD individually and in combination to identify the first E2 core domain (McCaffrey et al., 2007). While all three variable regions could be deleted without affecting CD81 binding, deletion of two of the three variable regions modulated CD81 binding function. Deletion of HVR2 and the igVR, or HVR1 and the igVR resulted in similar levels of CD81 binding as wild-type E2 RBD. However, deletion of HVR1 and HVR2 from the E2 RBD resulted in an approximately 50% reduction in CD81 binding. These results indicate that the presence of the igVR interferes with CD81 binding function when HVR1 and HVR2 is absent and point to a functional interaction between the igVR and HVR1 and/or HVR2 such that the CD81 binding site is properly formed or becomes fully accessible to the receptor. The available crystal structures of E2 are of a monomer. Absent from these structures is information about the E2 stem region, E1 and detailed structural information about HVR1, epitope I and HVR2. It is likely that the structure of intact E2 on the surface of a virion as a heterodimer with E1 differs from the structures of the E2 core thus far available. Indeed, all fusion proteins exist as either dimers of heterodimers or trimers of heterodimers and this is likely to be the case for HCV as well. Thus it is possible that in these higher order structures of virion incorporated E2, the variable regions may form additional contacts with the opposing E1 or E2 protomers providing additional mechanisms whereby HVR1, HVR2, and the igVR can interfere with antibody recognition and CD81 binding.

Finally, the reason why HVR2 and igVR sequences evolve rapidly in infected patients remains unresolved. Neither region has been implicated as being targets of the antibody response suggesting that a direct mechanism of immune evasion is not likely. However, it is possible that the sequence of HVR2 and the igVR could alter their ability to shield the E2 core from neutralizing antibodies or alters E1/E2 conformation while retaining function providing an allosteric mechanism of immune escape and require further studies.

## NEUTRALIZING AND NON-NEUTRALIZING ANTIBODIES

A subset of epitope II directed non-neutralizing antibodies have been reported to interfere with the capacity of epitope I antibodies to neutralize virus (Zhang et al., 2009) while other epitope II-directed NAbs act cooperatively with epitope I specific NAbs (Keck et al., 2012, 2013). The ability of neutralizing antibodies directed toward epitope II to prevent neutralization escape suggests that they are a highly desirable component of the antibody response to any HCV vaccine but it is not clear what proportions of antibodies specific to epitope II have neutralizing/non-neutralizing activity in natural infection or in vaccination. Analysis of the fine specificity of the antibody response in natural infection is necessary to dissect these effects and examine their neutralization capacity.

## GLYCOSYLATION OF E2 AND ITS EFFECT ON NEUTRALIZATION

E2 contains 11 sites for N-linked glycosylation that are largely conserved in different HCV isolates suggesting they play important roles in glycoprotein structure and function. Helle et al. (2007) found that Asn417, Asn532, and Asn623 obscured E2 from CD81 binding and interfered with the ability of antibody to neutralize virus in the context of HCVpp. In HCVcc, an additional two glycosylation sites impacted on neutralization sensitivity at Asn423 and Asn446 (Helle et al., 2010). All five glycosylation sites are directly adjacent to CD81 contact residues suggesting that they may restrict the ability to elicit NAbs toward these CD81 binding regions or impact on accessibility of underlying epitopes to antibody neutralization. In genotype 3a viruses, an additional glycosylation site at position 495 reduces the sensitivity of HCV to neutralization using pooled HCV positive immune serum, and suggests that in some viruses, additional mechanisms of glycan mediated immune escape may evolve (Anjum et al., 2013). The structure of E2 indicates that 7/11 glycans shield the neutralizing face of the E2 core domain confirming that glycans potentially have the ability to restrict the generation of antibodies to these regions in natural infection and vaccination and /or restrict antibody access to epitopes.

## IMPACT ON VACCINE DESIGN

While recombinant E2 provides an obvious vaccine candidate as it can be produced in large amounts, the above information suggests its use requires caution. Variable regions within E2 have the potential to occlude the underlying CD81 binding site, HVR1 is immunodominant and drives immune escape and the extensive glycan shield protects the neutralizing face of E2. In addition, the use of isolated E2 may promote the elicitation of antibodies to the non-neutralizing face highly exposed in monomeric E2. The lack of neutralizing ability of antibodies directed to this non-neutralizing face of E2 is likely to be explained by its occlusion in virion incorporated E1/E2 heterodimers or its occlusion in the higher order arrangement of E1/E2 heterodimers in the virion, e.g., dimers or trimers of heterodimers. These caveats on the use of E2 as an immunogen are highly reminiscent of the HIV-1 gp120 monomer that contains five variable regions, extensive glycan shielding, and an immunodominant non-neutralizing face.

The low frequency of antibodies elicited to epitope I in natural infection suggest it is subdominant and engineering of the E2 protein may be required to increase the titre of antibodies specific to this region in vaccine candidates. Similar studies investigating the titre and prevalence of antibodies directed toward epitopes II or III are required to understand whether these specificities are elicited in native forms of E2 or whether reengineering of E2 is required to make these epitopes immunodominant. Finally, key to successful vaccine design is an understanding of the correlates of protective immunity. Future studies aimed at delineating the fine specificity of the antibody response in people who successfully clear their HCV infection will provide essential information to guide the rational design of an HCV vaccine.

## Conflict of Interest Statement

The author declares that the research was conducted in the absence of any commercial or financial relationships that could be construed as a potential conflict of interest.
